# What's new in *SNI* for May, 2010?

**DOI:** 10.4103/2152-7806.63901

**Published:** 2010-05-31

**Authors:** James I Ausman

**Affiliations:** Editor-in Chief, Surgical Neurology International

Although papers in *Surgical Neurology International* (SNI) are published 2 weeks after acceptance, the Editors plan to write a monthly commentary on the papers that have been published, as a service to our readers. This feature, which involves an editorial perspective on the papers that are published, has been very popular in the past, as it places the various papers into a meaningful context.

In future papers, we will be publishing the comments of all the reviewers so that the reader can benefit from the different opinions expressed about the papers.

Also, new editorials will appear each month from many sources. This feature has always been popular with our readers. We will also have Guest Editorials on subjects outside the Neurosciences to provide our readers with a broader perspective of what is happening in the world around us.

The Editorial Board wants you to know that this is *your* journal. We are open to your ideas and contributions. We welcome operative videos, lectures, and contributions from all centers and people around the world. Obviously, the Editorial Board will look at all of these contributions for quality and value for our readers. We are open to controversy, as we always have been. We want to hear what you think, so we can find the truth.

For those who want to participate in this new venture, please write to me at jia@surgicalneurologyint.com and let me know what your ideas are to make this better and how you can help us reach that goal.

## MAY 2010

I have written an editorial, The beginning of *Surgical Neurology International* describing all the new features in the journal and Web site. There are many more on the way.

I have also written an editorial, “Welcome to the 21^st^ Century.” This editorial provides some understanding of the chaotic world in which we live, why it is so, and its meaning for all of us as we enter the 21^st^ century.

We expect to have papers on Translational Neuroscience – basic science written so that busy neurosurgeons and neurologists can understand it. Also, we will have papers submitted on new clinical research that is innovative and probes biological mysteries. Finally, we will have a section on Fundamental Neurosurgery. This section will have papers that come from all parts of the world that teach us fundamentals of neurosurgery and neurology.

This month we begin with a summary of five papers published in various basic and clinical medical science journals that deal with the molecular basis of medicine, neurosurgery, and neurology – Translational Neuroscience. The first is an excellent paper from *Lancet* describing how a patient's genome was used to predict diseases and drug sensitivities that the patient might experience in his future. This is a landmark paper in medicine and predicts how we will use genetic screening in the future. A second paper deals with the genetic and molecular mechanisms that lead to NF2 tumor formation. There is another interesting study that shows how the viral based transfer of genes to chondrocytes in an animal model of disc degeneration can rebuild a degenerated disc. A fourth paper deals with a molecular treatment that blocks a membrane receptor allowing water to enter cells after an infarct. This drug prevents the malignant cerebral edema that occurs after an infarct and avoids the need for a decompressive craniotomy in the animal model. A final paper deals with a new approach to obliterating aneurysms by delivering a liquid formation containing VGEF, an endothelial growth factor, to the aneurysm site so that cellular proliferation can be stimulated to obliterate the aneurysm. These notes are all written so that anyone in the world can understand the basic principles of the work and its implications for our practice.

Villavicencio *et al* from the United States (USA) have written one of the best studies I have read on a comparative approach to two surgical approaches to spine surgery using a minimally invasive (MI) and an open technique. The authors treated patients with “painful degenerative disk disease with or without disc herniation, spondylolisthesis and/or stenosis at 1 or 2 spinal levels.” Either an open or minimally invasive transforaminal lumbar interbody fusion (TLIF) was performed in a group of 63 or 76 patients respectively. The authors found that the long-term outcomes of each procedure were the same after surgery. They also measured operative time, blood loss, and duration of hospitalization. However, the neurological deficits in the minimally invasive surgery were 6.5 times higher (10.5% vs. 1.6%) in the open surgical group of patients although the length of stay and blood loss were lower in the minimally invasive group. Nancy Epstein, a spine surgery expert for *SNI*, comments: “*I have not seen any study which so vertically confirmed what we have all suspected. Furthermore, they documented a greater incidence of new postoperative neurological deficits in the MI vs. the open population. We have all been waiting to hear such findings, which are likely buried in other studies. In short, I commend the authors for an excellent study ‘well done’….*”

Nancy Epstein, from the USA and a member of the *SNI* Editorial Board and an Associate Editor, explores the human side of malpractice cases involving quadriplegia. After examining 54 cases, 15 of which had verdicts for the defense and no compensatory damages, she asks is this a fair outcome? Under a “No Fault” system the injured party would be compensated reasonably and quickly and the excess costs of the litigation would be avoided with the damages going to the patient. This is an excellent paper, which raises questions that we do not hear about this subject.

Kimmel *et al* from the USA have a case report on the occurrence of chordoma in the lateral medullary cistern in a patient with tuberous sclerosis. Although on the surface this paper appears to be a routine case report, its significance is in the genetic causes of the chordoma, which is a rare tumor and has been reported in association with tuberous sclerosis. The important part of this paper is in the Discussion, in which the genetics of this tumor are clearly presented. Only one other case in the literature has analyzed the genetic abnormalities in chordomas found in a patient with tuberous sclerosis with the same genetic abnormalities as in this reported case. Other chordomas analyzed, which where not associated with tuberous sclerosis, and reported in the literature, have “no consistent cytogenetic abnormality … in any of the chordomas” according to the authors. Read the paper on Translational Neuroscience describing the well-defined genetic abnormalities in NF2 patients and begin to draw a parallel with tuberous sclerosis. This is where medicine, neurology, and neurosurgery are going.

Xu *et al* from the USA describe a case of non-small cell lung cancer metastasis to the craniocervical junction. The patient was neurologically intact but had severe neck pain. Imaging showed lytic destruction of the occipital condyle with a fracture of the atlanto-occipital joint. The authors performed a fusion from the occiput to C5 with good results. The images and operative illustration are excellent. The patient lived a quality life for 14 months. Seven cases with other tumors have been reported in the literature and as one reviewer suggested it would be nice to have more cases, but this is a rare entity and that would take some time. The authors′ final sentence in the discussion is: *“As healthcare policies in the United States continue to evolve, it is hoped that the decision on whether to pursue an operative fusion procedure in the face of metastatic disease will remain a choice made jointly by the patient, his/her treating oncologist and neurosurgeon.”* This is the key question the paper poses.

Ramsis Ghaly, a practicing neurosurgeon, neurointensivist and neuroanesthesiologist, discusses an important issue in a Letter to the Editor. What is the best treatment for the patient with a ruptured intracranial aneurysm? What he says is true. Patients are automatically being directed to coiling without a consideration of what is best for the patient. Interventionalists obtain consent for an angiogram and coiling at the same time depriving the patient of a choice depending upon what the angiogram shows. The neurosurgeon is told that the patient is on the table and the aneurysm can be coiled immediately. Or the neurosurgeon is never called to discuss what treatment is best. I have seen this practice all over the USA. It is not common in the developing world where coils are expensive and interventionalists are uncommon. There needs to be a discussion between the neurosurgeon and the interventionalist about what is best for the patient and who can provide the treatment with the lowest risk and the best outcome. That is not happening. Is money possibly involved in this process? What do you think?

Prabbu *et al*, from the USA, describe a case of a large paracavernous mass that compresses the brain stem. There were no clinical signs related to this lesion. What would you do to treat this lesion located on the left side? Would you operate? What are the goals of your surgery? The preoperative diagnosis could not be determined by biopsy at another institution. What is the lesson here?

Ziewacz *et al*, from the USA, discuss a case of a patient who developed a tentorial osteosarcoma at the site of radiation for Hodgkin's Lymphoma. The patient presented 22 years later with the tentorial mass. It became widely metastatic. Read the Discussion, which summarizes the knowledge of this radiation-induced tumor. The chilling reminder is that radiation therapy is not benign and has a long course before neoplasia develops. We recently operated on a patient who developed cortical meningiomas 40 years after scalp radiation for tinea capitis, a treatment commonly used in the 1940s. Will we see the effects of radiation from CTs in the future? Patients must know this risk.

Pieter Kubben, a resident in the Netherlands, who is highly praised for his knowledge of Internet applications to medicine, has written an introductory editorial on the section he will edit for the Web site on Neurosurgery 2.0, leading all of us into the 21^st^ century in this rapidly-developing area. Read his editorial and look at his Web site contribution.

I invited Edward Gordon to write a guest editorial on the worldwide talent shortage that affects the field of medicine and neurosurgery. Ed is a world expert on this subject and has written a number of authoritative books in this field. He gives us a perspective on how this problem will be solved around the world.

Two important books are also reviewed; One, probably the best, most concise book in the field of neurosurgery, in my opinion, is Greenberg's 7^th^ edition of the *Handbook of Neurosurgery* and a second is a new 7.0 *Tesla MR Atlas of the Brain*.

